# Differential effects of glyoxalase 1 overexpression on diabetic atherosclerosis and renal dysfunction in streptozotocin‐treated, apolipoprotein E‐deficient mice

**DOI:** 10.14814/phy2.12043

**Published:** 2014-06-11

**Authors:** Michèle Geoffrion, Xueliang Du, Zehra Irshad, Barbara C. Vanderhyden, Kerri Courville, Guangzhi Sui, Vivette D. D'Agati, Sylvie Ott‐Braschi, Naila Rabbani, Paul J. Thornalley, Michael Brownlee, Ross W. Milne

**Affiliations:** 1 Atherosclerosis, Genetics and Cell Biology Group, University of Ottawa Heart Institute, Ottawa, Ontario, Canada; 2 Diabetes Research Center, Albert Einstein College of Medicine, Bronx, New York; 3 Clinical Sciences Research Laboratories, Warwick Medical School, University of Warwick, Coventry, U.K.; 4 Centre for Cancer Therapeutics, Ottawa Hospital Research Institute, Ottawa, Ontario, Canada; 5 Department of Pathology, Columbia University, New York, New York; 6 Department of Pathology and Laboratory Medicine, University of Ottawa, Ottawa, Ontario, Canada

**Keywords:** ApoE‐deficient mice, atherosclerosis, diabetes, glyoxalase, nephropathy

## Abstract

The reactive dicarbonyls, glyoxal and methylglyoxal (MG), increase in diabetes and may participate in the development of diabetic complications. Glyoxal and MG are detoxified by the sequential activities of glyoxalase 1 (GLO1) and glyoxalase 2. To determine the contribution of these dicarbonyls to the etiology of complications, we have genetically manipulated GLO1 levels in apolipoprotein E‐null (*Apoe*^*−/−*^) mice. Male *Apoe*^*−/−*^ mice, hemizygous for a human GLO1 transgene (GLO1TG*Apoe*^*−/−*^ mice) or male nontransgenic *Apoe*^*−/−*^ litter mates were injected with streptozotocin or vehicle and 6 or 20 weeks later, aortic atherosclerosis was quantified. The GLO1 transgene lessened streptozotocin (STZ)‐induced increases in immunoreactive hydroimidazolone (MG‐H1). Compared to nondiabetic mice, STZ‐treated GLO1TG*Apoe*^*−/−*^ and *Apoe*^*−/−*^ mice had increased serum cholesterol and triglycerides and increased atherosclerosis at both times after diabetes induction. While the increased GLO1 activity in the GLO1TG*Apoe*^*−/−*^ mice failed to protect against diabetic atherosclerosis, it lessened glomerular mesangial expansion, prevented albuminuria and lowered renal levels of dicarbonyls and protein glycation adducts. Aortic atherosclerosis was also quantified in 22‐week‐old, male normoglycemic *Glo1* knockdown mice on an *Apoe*^*−/−*^ background (*Glo1*KD*Apoe*^*−/−*^ mice), an age at which *Glo1*KD mice exhibit albuminuria and renal pathology similar to that of diabetic mice. In spite of ~75% decrease in GLO1 activity and increased aortic MG‐H1, the *Glo1*KD*Apoe*^*−/−*^ mice did not show increased atherosclerosis compared to age‐matched *Apoe*^*−/−*^ mice. Thus, manipulation of GLO1 activity does not affect the development of early aortic atherosclerosis in *Apoe*^*−/−*^ mice but can dictate the onset of kidney disease independently of blood glucose levels.

## Abstract (precis)

Increased levels of methylglyoxal and methylglyoxal‐derived advanced glycation end products may contribute to the development of diabetic complications. We show that overexpression of an enzyme that participates in the pathway of methylglyoxal detoxification, glyoxalase 1, protects streptozotocin‐treated, apolipoprotein E‐deficient mice from diabetic kidney disease but not from diabetes‐induced accelerated aortic atherosclerosis.

## Introduction

Diabetes‐induced metabolic derangements including elevated levels of advanced glycation end products (AGEs) are believed to play a causative role in the development of both microvascular and macrovascular diabetic complications (Vlassara and Palace 2002; Yan et al. 2003; Goldin et al. 2006). AGE levels are increased in diabetes (Thornalley et al. 2003) and immunoreactive AGEs are detected at sites of diabetic injury (Sugimoto et al. 1997; Hammes et al. 1999; Vlassara and Palace 2002; Forbes et al. 2003). AGE levels in skin are also strong predictors of cardiac mortality (Meerwaldt et al. 2007), retinopathy and nephropathy (Genuth et al. 2005) in diabetic patients. Reducing sugars and reactive dicarbonyls including glyoxal, methylglyoxal (MG), and 3‐deoxyglucosone can modify N‐terminal and lysine residue amino groups and arginine residue guanidino groups of protein to produce AGEs (Singh et al. 2001; Ulrich and Cerami 2001). MG concentrations are elevated 5‐ to 6‐fold in type 1 diabetic patients and 2‐ to 3‐fold in type 2 diabetic patients (Thornalley 1993) and, when quantified by liquid chromatography/tandem mass spectroscopy (LC‐MS/MS), MG‐derived hydroimidazolone, N_*δ*_‐(5‐hydro‐5‐methyl‐4‐imidazolon‐2‐yl)‐ornithine (MG‐H1), is the predominant AGE in tissues of diabetic humans and rats (Thornalley et al. 2003). MG can be generated by a number of metabolic pathways (Fu et al. 1996; Thornalley 1998; Januszewski et al. 2003; Deng and Yu 2004; Turk 2010), with triose phosphate intermediates of glycolysis being a major source of MG in diabetes (Brownlee 2001). In cells, MG is detoxified primarily by the glyoxalase system. In the presence of reduced glutathione (GSH), MG spontaneously forms a hemithioacetal. Glyoxalase I (GLO1) catalyzes the isomerization of the hemithioacetal to *S*‐D‐lactoylglutathione that is then converted to D‐lactate by glyoxalase II (Thornalley 1993, 1998; Thornalley 2003).

Methylglyoxal and MG‐derived AGEs could potentially interfere with normal cell function and contribute to pathology by several distinct mechanisms. Approximately 0.2–0.4% of arginine residues are modified by MG‐H1 adducts in normal rat tissues and this is doubled if the rats are rendered diabetic with streptozotocin (STZ; Thornalley et al. 2003). Mitochondrial proteins (Rabbani and Thornalley 2008; Wang et al. 2009), proteasomal subunits (Queisser et al. 2010), factors involved in transcriptional regulation (Yao et al. 2007; Ceradini et al. 2008; El‐Osta et al. 2008; Thangarajah et al. 2009) and enzymes that provide a defense against oxidative stress (Paget et al. 1998; Wu and Juurlink 2002; Wang et al. 2009), and dicarbonyl stress (Takahashi et al. 1995) are among the targets of AGE modification and their inactivation or impaired function could negatively affect cell physiology. MG is present in extracellular fluid and plasma and can arise from different sources (Brownlee 2001; Kalapos 2013). AGE cross‐linking of extracellular matrix proteins in the vessel wall increases vascular stiffness (Wolffenbuttel et al. 1998; Vasan et al. 2003) and can decrease cell adherence and modify integrin‐mediated signaling (Beltramo et al. 2002; Dobler et al. 2006; Bhatwadekar et al. 2008). AGE‐modified extracellular proteins may also cause injury via binding to cell surface AGE receptors, of which, the best characterized is RAGE, the Receptor for AGEs. While there are differing views on whether AGE‐modified proteins are pathophysiological ligands for RAGE (Ahmed and Thornalley 2007; Heizmann 2007; Ramasamy et al. 2007), it is clear from experiments with RAGE‐null mice that RAGE makes an important contribution to both macrovascular (Soro‐Paavonen et al. 2008) and microvascular (Myint et al. 2006; Toth et al. 2008; Reiniger et al. 2010) diabetic complications possibly by binding non‐AGE, RAGE ligands such as S100/calgranulins and high mobility group box 1 protein (HMGB1). Moreover, there is a complex interaction between RAGE and GLO1 gene expression in the context of hyperglycemia. Exposure of cultured endothelial cells to hyperglycemia upregulates expression of RAGE, S100B, and HMGB1 and this can be prevented by overexpression of GLO1 (Yao and Brownlee 2009), whereas genetic inactivation of RAGE prevents the diabetes‐induced downregulation of GLO1 expression in kidneys of OVE26 mice, a mouse model of type 1 diabetes (Reiniger et al. 2010). It has been recently shown that, in humans, compared to stable carotid atherosclerotic plaques, rupture‐prone plaques have decreased GLO1 mRNA and protein and increased protein‐bound MG‐H1 and N*ε*‐carboxymethyllysine (Hanssen et al. 2014).

Genetic manipulation of GLO1 levels in rodent models has provided additional support for the role of dicarbonyl stress in the development of diabetic complications. Increasing GLO1 activity by somatic gene transfer reduces diabetic hyperalgesia in STZ‐treated mice, whereas hyperalgesia is evident in nondiabetic GLO1 knockdown mice (Bierhaus et al. 2012). Overexpression of GLO1 in bone marrow cells can also reverse impaired neovascularization in diabetic mice after experimental hind limb ischemia (Vulesevic et al. 2014). The increased GLO1 activity in human GLO1 transgenic rats results in lower levels of dicarbonyls and dicarbonyl‐derived AGEs in blood and tissues (Brouwers et al. 2011), and protects against diabetes‐induced endothelial dysfunction (Brouwers et al. 2010) and retinal pathology (Berner et al. 2012). Renal senescence in nondiabetic GLO1 transgenic rats is also retarded. (Ikeda et al. 2012). We have recently shown that GLO1 knockdown (*Glo1*KD) mice have increased renal levels of MG‐H1 and 3‐nitrotyrosine (3‐NT), a marker of oxidative stress, and show structural and functional changes in the kidney that are similar to those of diabetic mice in spite of normal levels of blood glucose. In contrast, STZ‐treated GLO1 transgenic mice are protected from renal dicarbonyl and oxidative stress and from diabetes‐induced kidney pathology, despite hyperglycemia (Giacco et al. 2014). Thus, MG levels can determine the onset of kidney disease in mice independently of blood glucose levels. Here, we have examined if MG levels have a similar predominant role in the onset of diabetic macrovascular disease. We have tested if GLO1 overexpression can protect against accelerated atherosclerosis in STZ‐treated apolipoprotein E‐deficient (*Apoe*^*−/−*^) mice and if GLO1 knockdown can promote atherogenesis in nondiabetic *Apoe*^*−/−*^ mice.

## Research Design and Methods

### Mice and induction of diabetes

The production and preliminary characterization of the cmyc epitope‐tagged human GLO1 transgenic mice have been described previously (Giacco et al. 2014; Vulesevic et al. 2014). For the atherosclerosis and nephropathy studies reported here, the GLO1 transgenic mice were bred onto an *Apoe*^*−/−*^ background. Male mice hemizygous for the GLO1 transgene (GLO1TG*Apoe*^*−/−*^) were compared to male nontransgenic *Apoe*^*−/−*^ littermates (non‐TG mice). To induce diabetes, 10‐week‐old GLO1TG*Apoe*^*−/−*^ and non‐TG males were injected intraperitoneally with 50 mg/kg of streptozotocin (STZ) in 0.05 mol/L sodium citrate (pH 4.5) on five consecutive days. Control GLO1TG*Apoe*^*−/−*^ and non‐TG males were similarly injected with citrate buffer. One week later and, again, 1 day before the mice were sacrificed, blood was taken from the saphenous vein for measurement of blood glucose concentration (One‐Touch, Ultra glucometer). STZ‐injected GLO1TG*Apoe*^*−/−*^ and non‐TG mice with blood glucose levels above 12 mmol/L were considered diabetic and included in the study. Glo1 knockdown mice (El‐Osta et al. 2008; Giacco et al. 2014) were bred onto an *Apoe*^*−/−*^ background (*Glo1*KD*Apoe*^*−/−*^ mice) and were maintained on a standard chow diet. All animal procedures confirm to the Guide for the Care and Use of Laboratory Animals published by the US National Institutes of Health (NIH Publication No. 85‐23, revised 1996).

### Determination of immunoreactive human GLO1, GLO1 activity, and reduced glutathione in cell and tissue extracts

Immunoreactive cmyc‐hGLO1 was detected in cell and tissue extracts by western blotting using the anti‐cmyc monoclonal antibody 9E10. The 9E10 hybridoma was obtained from ATCC. GLO1 activity in cell and tissue samples was measured by the method of Oray and Norton (Oray and Norton 1982). Reduced glutathione was measured using the Promega GSH‐Glo^™^ glutathione assay kit (Promega, Madison, WI) using the protocol provided by the manufacturer.

### Quantification and characterization of atherosclerotic lesions

The method for quantification of atherosclerotic lesions in tissue sections of the aortic root has been described in detail (Daugherty and Whitman 2003). Briefly, lesion size in the ascending aorta was determined from four 10‐*μ*m Oil Red O‐stained serial sections, taken at 100‐*μ*m intervals with the first section (level 0) being defined as that including the ostia for the coronary arteries. Lesion area was determined as intimal tissue within the internal elastic lamina quantified using Image‐Pro software (Media Cybernetics). The lesion complexity index in the aortic root was determined according to the criteria of the Animal Models of Diabetic Complications Consortium (http://www.amdcc.org). Briefly, on the same slide, the ratio of complex lesions (with cholesterol clefts, necrosis, or fibrous cap formation) to total lesions was determined. Aortic tissues were prepared for *en face* analysis as has been described (Daugherty and Whitman 2003). Digital images of the exposed intimal surface were captured and analyzed using Image Pro software. Macrophages were identified in frozen sections of the ascending aorta using a rat anti‐mouse CD68 primary antibody (Serotec #MCA1957) followed by sequential incubations with a horse radish peroxidase (HRP)‐conjugated goat anti‐rat‐IgG antibody (Serotec #STAR72) and the HRP substrate, 3,3′diaminobenzidine (Vector Laboratories).

### Plasma lipid concentrations and lipoprotein profiles

Serum cholesterol and triglyceride concentrations were measured using commercial kits (Wako Bioproducts, Richmond, VA). To determine serum lipoprotein profiles, 50‐*μ*L serum samples from individual mice were subjected to Superose 6 (Pharmacia LKB Biotechnology, Uppsala, Sweden) gel exclusion chromatography and total cholesterol concentrations (Wako Bioproducts) were measured in 0.5 mL fractions.

### Determination of dicarbonyls and protein glycation adducts

Methylglyoxal content of kidney samples was assayed by stable isotopic dilution analysis liquid chromatography–tandem mass spectrometry (LC‐MS/MS) as described (Kurz et al. 2011). Protein glycation adducts and the nitration marker 3‐nitrotyosine (3‐NT) were detected and quantified by stable isotopic dilution analysis LC‐MS/MS. Kidney soluble protein extracts were prepared, hydrolyzed enzymatically, and assayed as described (Karachalias et al. 2010). Protein glycation adducts of aortal collagen was assayed similarly with a modified enzymatic hydrolysis employing bacterial collagenase (Dobler et al. 2006). Protein glycation adducts determined were as follows: the early‐stage glucose‐derived glycation adduct N*ε*‐fructosyl‐lysine (FL) and AGEs: MG‐derived AGEs – hydroimidazolone MG‐H1 and N*ε*‐carboxyethyl‐lysine (CEL); glyoxal‐derived AGEs – hydroimidazolone G‐H1, N*ε*‐carboxymethyl‐arginine (CMA), and N*ε*‐carboxymethyl‐lysine (CML; also formed from degradation of FL); and others – 3‐DG‐derived hydroimidazolones (3DG‐H) and pentosidine. Detection of immunoreactive MG‐HI in tissues by immunohistochemistry using a anti‐MG‐H1 monoclonal antibody (Kilhovd et al. 2003) was performed as described previously (Giacco et al. 2014). Detection and quantification of MG‐H1‐modified proteins by immunoprecipitation and western blotting has been described in detail (Thangarajah et al. 2009).

### Assessment of albuminuria and renal pathology

At week 20, mice were placed in metabolic cages for 24 h and urine was collected. Urinary albumin was measured as described (Giacco et al. 2014). Severity of mesangial sclerosis was based on the mesangial area occupied by matrix and was scored on a semiquantitative 0–3 scale by a renal pathologist (V. D'Agati) blinded to the genotype and treatment protocol of the mice according to the previously published method (Reiniger et al. 2010).

### Cytokine arrays

Thirty‐two cytokines were measured in 50 *μ*g of aortic extracts from mice using the RayBio^®^ Mouse Cytokine Antibody Array G2 (Ray Biotech Inc., Norcross, GA) according to the protocol provided by the manufacturer. Fluorescence was measured with a GenePix 4000B Microarray Scanner (Molecular Devices, Sunnyvale, CA).

## Results

### Characterization of human GLO1 transgenic mice

**Figure 1.**
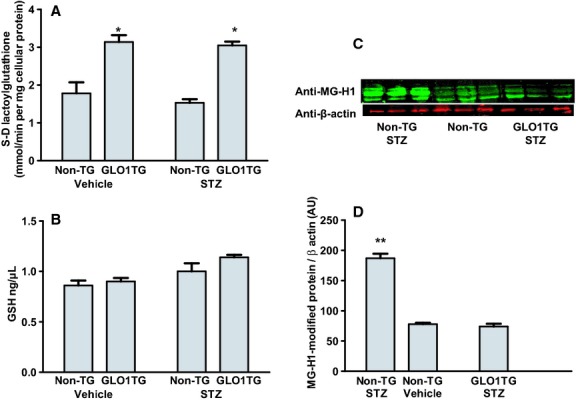
Glyoxalase 1 (GLO1) activity, reduced glutathione (GSH), and MG‐H1 immunoreactivity in aortic extracts of GLO1TG mice and nontransgenic (Non‐TG) littermates that had been treated with streptozotocin (STZ) or vehicle. Mice were administered STZ or vehicle and 6 weeks later they were sacrificed and GLO1 activity (A), GSH levels (B) and MG‐H1 immunoreactivity (C and D) in aortic extracts were determined. *P < 0.001 GLO1TG versus nontransgenic, **P < 0.0001 STZ‐treated nontransgenic mice versus either nontransgenic vehicle‐treated mice or STZ‐treated GLO1TG mice.

As we have reported previously, immunoreactive cmyc‐GLO1 was detected by western blotting in all tissues from GLO1TG mice that were tested (heart, aorta, kidney, liver, eye, and brain) and extracts from these tissues showed an approximately 1.5‐fold higher GLO1 activity compared to the respective tissues from nontransgenic littermates. Immunoreactive cmyc‐GLO1 was detected in cultured aortic endothelial cells, in aortic smooth muscle cells and in peritoneal and bone marrow‐derived macrophages isolated from GLO1TG mice and there was an approximately 5‐fold higher GLO1 activity in the cells from the GLO1TG mice compared to the equivalent cells isolated from nontransgenic littermates (Vulesevic et al. 2014). To test the effect of diabetes on GLO1 activity and the efficacy of the GLO1 transgene in reducing MG modification of proteins, GLO1TG mice and nontransgenic litter mates were administered STZ or vehicle. As was the case for nondiabetic mice, 6 weeks after induction of diabetes, GLO1 activity in aortic extracts from GLO1TG mice was about 1.5‐fold that of non‐TG mice (Fig. 1A). Neither STZ treatment, the presence of the GLO1 transgene (Fig. 1B) nor the administration of N‐acetylcysteine in the drinking water (not shown) significantly altered reduced glutathione levels in the aorta. Notably, the elevated GLO1 activity in the GLO1TG mice prevented the increase in MG‐H1 immunoreactive proteins in aortas (Fig. 1C and D) and kidneys (not shown) of the STZ‐treated mice. For all experiments described below, GLO1TG mice were bred onto an *Apoe*^*−/−*^ background.

### Effect of the *hGlo1* transgene on atherosclerosis in diabetic *Apoe*^*−/−*^ mice

**Figure 2.**
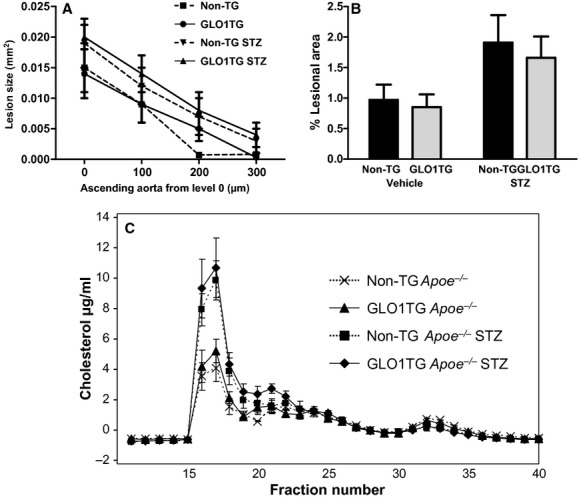
Early atherosclerosis in the aortic root (A) and aortic arch (B) of diabetic and nondiabetic non‐TG*Apoe*^*−/−*^ mice (non‐TG) and GLO1TG*Apoe*^*−/−*^ (GLO1TG) mice. Ten‐week‐old non‐TG*Apoe*^*−/−*^ or GLO1TG*Apoe*^*−/−*^ mice were injected with either streptozotocin (STZ) or vehicle as described in Research Design and Methods and were sacrificed 6 weeks later. Atherosclerosis was quantified in the ascending aorta at four levels separated by 100 *μ*m and, by *en face* analysis, in the aortic arch. Atherosclerosis in the aortic root was not significantly affected by either STZ treatment or genotype. Diabetes significantly increased lesion area in the aortic arch (*P* = 0.02) and this was independent of the GLO1 transgene. (C) Cholesterol distribution in serum lipoprotein subfractions of mice 6 weeks after administration of STZ or vehicle. Serum from individual mice (*n* = 5) was subjected to Superose 6 gel exclusion chromatography. Cholesterol levels were determined in the column fractions.

It has been reported that injection of *Apoe*^*−/−*^ mice with STZ leads to accelerated atherosclerosis that is evident as early as 6 weeks after diabetes induction (Park et al. 1998). To test if increased GLO1 activity can retard the development of diabetic atherosclerosis, 10‐week‐old GLO1TG*Apoe*^*−/−*^ male mice and non‐TG *Apoe*^*−/−*^ male litter mates maintained on a chow diet were injected with STZ or vehicle and, 6 weeks later, the animals were sacrificed and aortic atherosclerosis was quantified. Over the 6 weeks, both Glo1TG*Apoe*^*−/−*^ and non‐TG*Apoe*^*−/−*^, STZ‐treated mice gained weight more slowly than did their nondiabetic counterparts. The GLO1 transgene did not influence fasting glucose levels. At the time of sacrifice, plasma cholesterol levels in STZ‐treated mice were about twice those of the vehicle‐injected mice (Table 1), with the excess cholesterol being associated primarily with VLDL/LDL‐sized lipoproteins (Fig. 2C) and neither plasma cholesterol levels nor lipoprotein profile were significantly altered by the GLO1 transgene. Plasma triglycerides were also higher in the diabetic mice and did not differ between GLO1TG*Apoe*^*−/−*^ mice and non‐TG mice. No differences between groups were observed in the level of the GLO1 cofactor, reduced GSH, in heart tissue adjacent to the aortic root (Table 1). As is seen in Figure 2A, mice showed little atherosclerosis in the aortic root and, while GLO1TG*Apoe*^*−/−*^ mice and non‐TG*Apoe*^*−/−*^ littermates that had been administered STZ, showed a trend toward increased lesion area compared to vehicle‐treated mice, this did not achieve statistical significance (*P* < 0.1). No differences in aortic root lesions between diabetic GLO1TG*Apoe*^*−/−*^ and diabetic nontransgenic litter mates were observed. *En face* analysis of atherosclerosis in the aortic arch (Fig. 2B) showed that lesion areas were similar between diabetic GLO1TG*Apoe*^*−/−*^ and diabetic nontransgenic *Apoe*^*−/−*^ mice and, in both cases, were greater than nondiabetic mice (*P* < 0.02). Cytokine arrays on aortic extracts (*n* = 4) revealed no significant differences between any of the groups when normalized for total protein (not shown). Thus, in spite of increased GLO1 activity in tissues (Table 1), the GLO1TG*Apoe*^*−/−*^ mice were not protected from accelerated atherosclerosis due to the induction of diabetes in the aortic arch.

**Table 1.** Physical and biochemical characteristics of mice at time of sacrifice

Diabetes	Non‐TG*Apoe*^*−/−*^	GLO1TG*Apoe*^*−/−*^	STZ‐non‐TG *Apoe*^−/−^	STZ‐GLO1TG *Apoe*^*−/−*^
6 weeks				
*n*	14	9	14	15
Body weight	33.7 ± 1.0*	31.5 ± 1.2	26.9 ± 1.0	28.5 ± 1.1
Glucose (mmol/L)*	8.2 ± 0.5	7.2 ± 0.7	25.6 ± 1.2	26.6 ± 1.2
Cholesterol (mmol/L)*	9.1 ± 0.6	9.4 ± 0.7	17.7 ± 2.0	17.5 ± 1.3
Triglyceride (mmol/L)*	1.3 ± 0.1	1.0 ± 0.1	2.1 ± 0.4	1.6 ± 0.4
Glo1 activity* heart*	1.4 ± 0.1	2.1 ± 0.1	1.3 ± 0.1	2.1 ± 0.1
GSH (ng/*μ*L) heart*	7.8 ± 0.2	7.9 ± 1.0	7.9 ± 1.3	7.1 ± 0.3
20 weeks				
*n*	13	13	15	14
Body weight (g)	40.2 ± 2.0	37.8 ± 1.4	31.8 ± 0.7	30.5 ± 0.7
Glucose (mmol/L)*	8.1 ± 0.6	6.4 ± 0.4	26.8 ± 0.7	27.1 ± 1.1
Cholesterol (mmol/L)*	9.8 ± 0.7	12.0 ± 0.9	21.6 ± 1.3	23.3 ± 1.4
Triglyceride (mmol/L)*	0.6 ± 0.1	1.0 ± 0.2	1.6 ± 0.3	2.0 ± 0.4
GLO1 activity heart*	1.9 ± 0.6	3.2 ± 0.6	1.5 ± 0.1	2.6 ± 0.3
GLO1 activity kidney*	1.4 ± 0.1	1.6 ± 0.1	1.3 ± 0.1	1.8 ± 0.1
GSH (ng/*μ*L) heart*	9.0 ± 0.6	8.2 ± 0.5	9.0 ± 1.2	9.2 ± 0.3
GSH (ng/*μ*L) kidney*	8.9 ± 0.5	12.8 ± 4.0	10.7 ± 2.3	9.1 ± 0.4

* Mean ± standard error.

* S‐D‐lactoylglutathione formation (mmol/min/mg cellular protein).

* *P* < 0.0001 STZ‐treated versus vehicle‐treated (two‐way analysis of variance, ANOVA).

* *P* < 0.04 STZ‐treated versus vehicle‐treated (two‐way ANOVA).

* *P* < 0.0001 GLO1TG mice versus nontransgenic littermates (two‐way ANOVA).

* Normalized to cytosolic protein. See Research Design and Methods.

* *P* < 0.001 STZ‐treated versus vehicle‐treated (two‐way ANOVA).

* *P* < 0.02 GLO1TG mice versus nontransgenic littermates (two‐way ANOVA).

* *P* < 0.003 GLO1TG mice versus nontransgenic littermates (two‐way ANOVA).

**Figure 3.**
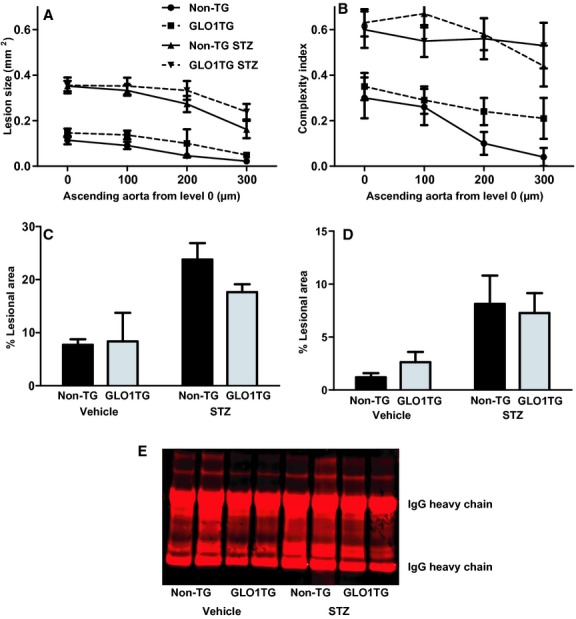
Late aortic atherosclerosis and MG‐H1 immunoreactivity in diabetic and nondiabetic GLO1TG*Apoe*^*−/−*^ (GLO1TG) mice and nontransgenic *Apoe*^*−/−*^ littermates (non‐TG). Ten‐week‐old non‐TG*Apoe*^*−/−*^ or GLO1TG*Apoe*^*−/−*^ mice were injected with either streptozotocin (STZ) or vehicle and were sacrificed 20 weeks later. Atherosclerotic lesion area (A) and lesion complexity (B) were quantified in the ascending aorta at four levels separated by 100 *μ*m. Atherosclerotic lesion area was quantified in the aortic arch (C) and descending thoracic aorta (between the origins of intercostal arteries 1 and 5) (D) by *en face* analysis. Diabetes significantly increased lesion area in the ascending aorta (*P* < 0.0001), the aortic arch (*P* < 0.0003), and the descending thoracic aorta (*P* < 0.0001) and this was independent of the GLO1 transgene. (E) MG‐H1 immunoreactivity in aortic extracts determined by immunoprecipitation/western blotting.

**Figure 4.**
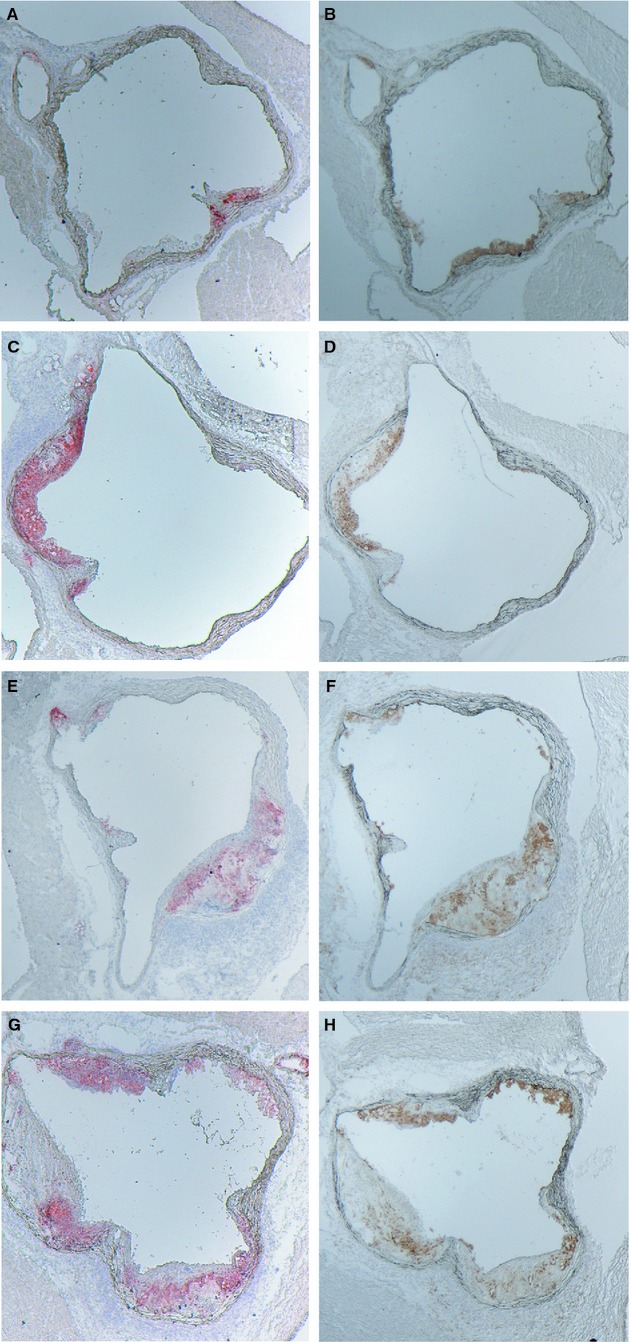
Lipid and macrophage accumulation in lesions of the aortic root. Sequential sections of the aortic root (situated between level 0 and 1) stained for lipid with Sudan IV (A, C, E, and G) or for CD68‐positive macrophages (B, D, F, and H) from GLO1TG*Apoe*^*−/−*^ mice (C, D, G, and H) or nontransgenic *Apoe*^*−/−*^ litter mates (A, B, E, and F) that had been administered streptozotocin (E–H) or vehicle (A–D).

At 6 weeks following induction of diabetes, we observed little atherosclerosis in the aortic root and aortic arch of the mice and the lesions that were present in the aortic root consisted predominantly of fatty streaks (not shown) and complex lesions were very rare. To test if increased GLO1 activity can prevent the further progression of diabetic atherosclerosis and the development of complex lesions, male GLO1TG*Apoe*^*−/−*^ and non‐TG*Apoe*^*−/−*^ litter mates were injected with STZ or vehicle and were maintained on a chow diet for 20 weeks before sacrifice. As was the case at 6 weeks after STZ administration, cholesterol and triglyceride levels were higher in the diabetic mice regardless of genotype (Table 1) with the cholesterol being primarily in the VLDL/IDL fractions (not shown). Quantification of lesions in the aortic root (Fig. 3A) showed that diabetes increases atherosclerosis in the mice and this is not mitigated by the GLO1 transgene. GLO1TG*Apoe*^*−/−*^ diabetic mice also did not differ from diabetic non‐TG*Apoe*^*−/−*^ mice in the numbers of complex lesions in the aortic root (Fig. 3B). Lesions were larger in GLO1TG*Apoe*^*−/−*^ (Fig. 4G) and nontransgenic litter mates (Fig. 4E) that had been administered STZ compared to the respective vehicle‐injected controls (Fig. 4A and C) and showed extensive macrophage infiltration (Fig. 4F and H). Likewise, the GLO1 transgene did not prevent the STZ‐induced increase in atherosclerosis in either the aortic arch (Fig. 3C) or the descending thoracic aorta between the origins of intercostal arteries 1 and 5 (Fig. 3D). Although the GLO1 transgene did not protect *Apoe*^*−/−*^ mice from accelerated atherosclerosis after STZ treatment, it appeared to reduce MG‐H1 immunoreactivity in aortic extracts from diabetic animals (Fig. 3E).

To gain further insight on the effect of the GLO1 transgene on glycation of aortal extracellular matrix (ECM), we analyzed protein glycation markers in aortal collagen of *Apoe*^*−/−*^ mice after 6 weeks and 20 weeks with and without STZ‐induced diabetes (Table 2). At 6 weeks the only changes in proteins glycation were an approximately 2‐fold increase of FL residue and 3‐fold increase in 3DG‐H residue content in STZ diabetic non‐TG*Apoe*^*−/−*^ with respect to nondiabetic controls which was not changed significantly by the GLO1 transgene. At 20 weeks, there was a further increase in FL residue content to 14.7 mmol/mol lys, 3‐fold higher than nondiabetic controls. 3DG‐H residue content of STZ diabetic *Apoe*^*−/−*^ mice remained increased by approximately 3‐fold and CML and MG‐H1 residue contents were also increased, 54% and 38%, respectively, in STZ diabetic *Apoe*^*−/−*^ mice. The GLO1 transgene did not prevent this increase.

**Table 2.** Protein damage of aortal collagen in *Apoe*^*−/−*^ mice – effect of the GLO1 transgene and streptozotocin‐induced diabetes

Analyte	Non‐TG*Apoe*^*−/−*^	GLO1TG*Apoe*^*−/−*^	*STZ‐*non‐TG*Apoe*^*−/−*^	*STZ‐*GLO1TG*Apoe*^*−/−*^
6 weeks				
FL (mmol/mol lys)	3.44 ± 0.76	3.42 ± 0.45	7.92 ± 1.45*	7.75 ± 1.70*^†^
CML (mmol/mol lys)	0.236 ± 0.026	0.216 ± 0.025	0.216 ± 0.020	0.206 ± 0.014
3DG‐H (mmol/mol arg)	0.074 ± 0.028	0.047 ± 0.013	0.216 ± 0.020*	0.206 ± 0.014*‡
MG‐H1 (mmol/mol arg)	0.358 ± 0.022	0.331 ± 0.025	0.327 ± 0.013	0.395 ± 0.037
CEL (mmol/mol lys)	0.066 ± 0.011	0.071 ± 0.009	0.045 ± 0.007	0.056 ± 0.012
CMA (mmol/mol arg)	0.124 ± 0.011	0.134 ± 0.017	0.154 ± 0.020	0.103 ± 0.023
Pentosidine (mmol/mol lys)	0.013 ± 0.009	0.007 ± 0.003	0.017 ± 0.001	0.017 ± 0.002
3‐NT (mmol/mol tyr)	0.054 ± 0.012	0.046 ± 0.007	0.042 ± 0.008	0.051 ± 0.012
20 weeks				
FL (mmol/mol lys)	4.92 ± 0.67	5.22 ± 0.62	14.70 ± 1.65***	14.70 ± 1.65***‡
CML (mmol/mol lys)	0.246 ± 0.037	0.237 ± 0.015	0.378 ± 0.046*	0.408 ± 0.042*‡
3DG‐H (mmol/mol arg)	0.076 ± 0.016	0.061 ± 0.011	0.190 ± 0.028**	0.171 ± 0.022**
MG‐H1 (mmol/mol arg)	0.407 ± 0.028	0.354 ± 0.010	0.560 ± 0.061*	0.486 ± 0.025‡
CEL (mmol/mol lys)	0.090 ± 0.007	0.102 ± 0.011	0.061 ± 0.012	0.088 ± 0.011
CMA (mmol/mol arg)	0.131 ± 0.008	0.127 ± 0.005	0.118 ± 0.013	0.130 ± 0.013
Pentosidine (mmol/mol lys)	0.025 ± 0.005	0.030 ± 0.003	0.024 ± 0.004	0.027 ± 0.006
3‐NT (mmol/mol tyr)	0.010 ± 0.002	0.012 ± 0.003	0.013 ± 0.004	0.012 ± 0.002

Data are mean ± SEM, *n* = 8.

Significance: *, ** and ***P < 0.05, *P* < 0.01 and *P* < 0.001 with respect to *Apoe*^*−/−*^ control; ^†^*P* < 0.05 compared to GLO1TG*Apoe*^*−/−*^ and ^‡^*P* < 0.01 compared to GLO1TG*Apoe*^*−/−*^.

### Effect of the *hGlo1* transgene on renal function in diabetic *Apoe*^*−/−*^ mice

**Figure 5.**
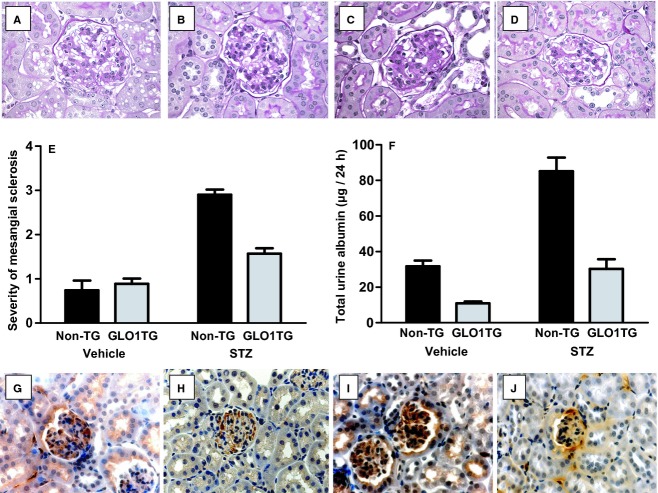
Mesangial sclerosis, albuminuria, and MG‐H1 immunoreactivity in diabetic and nondiabetic GLO1TG*Apoe*^*−/−*^ (GLO1TG) and in nontransgenic *Apoe*^*−/−*^ litter mates (non‐TG). Ten‐week‐old GLO1TG*Apoe*^*−/−*^ or non‐TG*Apoe*^*−/−*^ mice were injected with either streptozotocin (STZ) or vehicle and 20 weeks later mice were placed in metabolic cages, urine was collected over 24 h before the mice were sacrificed. Paraffin sections of renal cortex from GLO1TG*Apoe*^*−/−*^ (B, D, H, and J) and nontransgenic *Apoe*^*−/−*^ littermates (A, C, G, and I) treated with STZ (C, D, I, and J) or vehicle (A, B, G, and H) and were stained with periodic acid‐Schiff (A–D) or for MG‐H1 immunoreactivity with the 1H7C6 anti‐MG‐H1 monoclonal antibody (G–J). The severity of mesangial sclerosis was scored on a scale of 1–3 as described in the Research Design and Methods section (E) and showed that, in STZ‐treated mice, the GLO1 transgene significantly reduced the mesangial area occupied by matrix (*P* < 0.01). The GLO1 transgene also protected *Apoe*^*−/−*^ mice from the STZ‐induced increase in albuminuria (*P* < 0.001) (F).

Renal pathology and albuminuria were also evaluated 20 weeks after the GLO1TG*Apoe*^*−/−*^and non‐TG*Apoe*^*−/−*^ litter mates had been injected with STZ or vehicle. Mesangial sclerosis was apparent in diabetic non‐TG*Apoe*^*−/−*^ mice and this was significantly reduced in STZ‐treated GLO1TG*Apoe*^*−/−*^ mice (Fig. 5A–E). Urinary albumin excretion over 24 h in diabetic non‐TG*Apoe*^*−/−*^ mice was approximately 4‐fold that of nondiabetic, non‐TG*Apoe*^*−/−*^mice (Fig. 5F). Albumin excretion in the STZ‐treated GLO1TG*Apoe*^*−/−*^ mice did not exceed that of nondiabetic non‐TG*Apoe*^*−/−*^ mice. Nondiabetic GLO1TG*Apoe*^*−/−*^ mice also showed a significant decrease in albuminuria compared to that of nondiabetic, non‐TG*Apoe*^*−/−*^ mice. Measurement of methylglyoxal and markers of protein damage in renal tissue by LC‐MS/MS showed that diabetes increases MG, N*ε*‐carboxyethyllysine (CEL) and 3‐nitrotyrosine (3‐NT) levels in kidney homogenates of non‐TG*Apoe*^*−/−*^ mice with the diabetes‐induced increases being reduced or absent in kidney extracts from diabetic GLO1TG*Apoe*^*−/−*^ mice (Table 3). Fructosyllysine levels were significantly higher in STZ‐treated mice and this was independent of the GLO1 transgene. Based on the LC‐MS/MS results neither MG‐H1 nor CML levels were higher in diabetic mice and were not modulated by the higher GLO1 activity of the GLO1TG*Apoe*^*−/−*^ mice. However, by immunohistochemistry using an anti‐MG‐H1 monoclonal antibody, the renal cortex of STZ‐treated non‐transgenic mice (Fig. 5J) showed higher immunoreactivity than that of nondiabetic mice (Fig. 5G and H) or STZ‐treated GLO1TG*Apoe*^*−/−*^ mice (Fig. 5I). For all of the mice, the MG‐H1 immunoreactivity was especially evident in the glomeruli.

**Table 3.** Methylglyoxal and protein glycation in kidneys of GLO1TG*Apoe*^*−/−*^ and nontransgenic littermates, 20 weeks following completion of treatment with streptozotocin (STZ) or vehicle

	Non‐TG*Apoe*^*−/−*^	GLO1TG*Apoe*^*−/−*^	STZ‐non‐TG*Apoe*^*−/−*^	STZ‐GLO1TG*Apoe*^*−/−*^
MG (pmol/mg tissue wet weight)	3.24 ± 0.23*	3.45 ± 0.26	4.41 ± 0.38*	3.36 ± 0.18*
MG‐H1 (mmol/mol arg)	0.162 ± 0.011	0.173 ± 0.013	0.149 ± 0.013	0.155 ± 0.012
CEL (mmol/mol lys)	0.022 ± 0.004	0.015 ± 0.004	0.051 ± 0.011*	0.029 ± 0.004*
CML (mmol/mol lys)	0.141 ± 0.017	0.128 ± 0.020	0.160 ± 0.019	0.164 ± 0.020
FL (mmol/mol lys)	1.36 ± 0.14	1.82 ± 0.53	5.19 ± 0.69*	5.82 ± 0.36*
3‐NT (*μ*mol/mol tyr)	1.55 ± 0.54	0.92 ± 0.29	5.75 ± 1.52*	3.81 ± 0.48*

* Mean ± SEM.

* *P* < 0.03 non‐TG*Apoe*^*−/−*^STZ treated versus non‐TG*Apoe*^*−/−*^ vehicle treated.

* *P* < 0.04 GLO1TG*Apoe*^*−/−*^ STZ treated versus non‐TG*Apoe*^*−/−*^STZ treated.

* *P* < 0.003 non‐TG*Apoe*^*−/−*^STZ treated versus non‐TG*Apoe*^*−/−*^ vehicle treated.

* *P* < 0.05 GLO1TG*Apoe*^*−/−*^STZ treated versus non‐TG*Apoe*^*−/−*^STZ treated.

* *P* < 0.0001 non‐TG*Apoe*^*−/−*^STZ treated versus non‐TG*Apoe*^*−/−*^ vehicle treated.

* *P* < 0.02 h GLO1TG*Apoe*^*−/−*^STZ treated versus non‐TG*Apoe*^*−/−*^STZ treated.

### Effect of reduced GLO1 activity on atherosclerosis in nondiabetic *Apoe*^*−/−*^ mice

**Figure 6.**
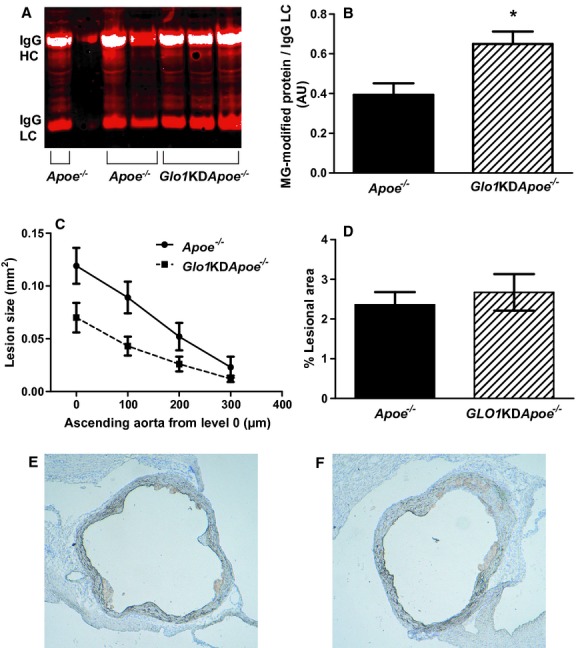
Immunoreactive MG‐H1‐modified proteins and atherosclerosis in the aortas of 22‐week‐old *Glo1*KD*Apoe*^*−/−*^ mice and nontransgenic *Apoe*^*−/−*^ littermates. MG‐H1 immunoreactive proteins were detected in aortic extracts with an MG‐H1‐specific monoclonal antibody (A) and were quantified and normalized to the light chain of the mouse IgG (IgG LC) used for immunoprecipitation (B). Atherosclerosis was quantified in the ascending aorta at four levels separated by 100 *μ*m (C) and, by *en face* analysis, in the aortic arch (D). Sections of the aortic root (situated between level 0 and 1) from nontransgenic *Apoe*^*−/−*^ (E) or *Glo1*KD*Apoe*^*−/−*^ (F) mice stained for CD68‐positive macrophages. *P < 0.04.

It is possible that the STZ‐induced increase in serum cholesterol in the GLO1TG*Apoe*^*−/−*^ may have masked any anti‐atherogenic effects of reduced dicarbonyl stress. To further explore if increased reactive dicarbonyl levels alone can exacerbate atherosclerosis in *Apoe*^*−/−*^ mice, we have bred *Glo1*KD mice that have a 45–65% reduction in GLO1 activity (El‐Osta et al. 2008) onto an *Apoe*^*−/−*^ background (*Glo1*KD*Apoe*^*−/−*^mice). The mice were maintained on a chow diet and, at 22 weeks of age, the *Glo1*KD*Apoe*^*−/−*^and age‐matched *Apoe*^*−/−*^ mice were sacrificed for quantification of atherosclerosis. At 22 weeks of age, *Glo1*KD mice show structural changes in the kidney similar to diabetic mice and increased albumin excretion compared to age‐matched wild‐type mice (Giacco et al. 2014). At the time of sacrifice *Glo1*KD*Apoe*^*−/−*^ and *Apoe*^*−/−*^ controls did not differ in terms of fasting serum glucose, cholesterol, or triglyceride levels and, in spite of GLO1 activities in tissues of *Glo1*KD*Apoe*^*−/−*^ mice that were approximately 25% of those in tissues of *Apoe*^*−/−*^ mice (Table 4). No differences were observed between *Glo1*KD*Apoe*^*−/−*^ mice and *Apoe*^*−/−*^ mice in the distribution of cholesterol among the serum lipoprotein subfractions (not shown). Reduced GLO1 activity in the *Glo1*KD*Apoe*^*−/−*^ mice increased MG‐H1 immunoreactivity in extracts of aorta (Fig. 6A and B) and kidney (not shown); however, the *Glo1*KD*Apoe*^*−/−*^mice did not develop more atherosclerosis at 22 weeks of age in either the aortic root (Fig. 6C, E and F) or the aortic arch (Fig. 6D) and actually showed a trend toward decreased lesion area in the aortic root (mean average lesion area calculated from the four transverse sections, *Glo1*KD*Apoe*^*−/−*^: 0.038 ± 0.008, *Apoe*^*−/−*^: 0.071 ± 0.013, *P* = 0.06). Measurement of aortal collagen glycation at 22 weeks revealed no significant differences between the *Glo1*KD*Apoe*^*−/−*^ and *Apoe*^*−/−*^ mice (Table 5).

**Table 4.** Physical and biochemical characteristics of mice at time of sacrifice

	20‐week *Apoe*^*−/−*^	20‐week *Glo1*KD*Apoe*^*−/−*^
Body weight (g)	31.1 ± 0.5	30.9 ± 0.4*
Glucose (mmol/L)	10.5 ± 0.9	10.3 ± 0.9
Cholesterol (mmol/L)	15.3 ± 0.9	15.6 ± 1.1
Triglyceride (mmol/L)	1.1 ± 0.1	1.3 ± 0.2
GLO‐1 activity heart*	1.18 ± 0.05	0.24 ± 0.04*
GLO‐1 activity kidney*	0.92 ± 0.04	0.24 ± 0.05*

* Mean ± standard error.

* S‐D‐lactoylglutathione formation (mmol/min/mg cellular protein).

* *P *< 0.0001 unpaired Student's *t*‐test.

**Table 5.** Glycation of aortal collagen in *Apoe*^*−/−*^ mice – effect of reduced GLO1 activity

Age	Analyte	*Apoe* ^*−/−*^	*Glo1*KD*Apoe*^*−/−*^
22 weeks	FL (mmol/mol lys)	5.24 ± 1.01	6.42 ± 0.86
	CML (mmol/mol lys)	0.430 ± 0.042	0.385 ± 0.048
	3DG‐H (mmol/mol arg)	0.060 ± 0.011	0.066 ± 0.019
	MG‐H1 (mmol/mol arg)	0.344 ± 0.034	0.375 ± 0.052
	CEL (mmol/mol lys)	0.155 ± 0.011	0.191 ± 0.019
	CMA (mmol/mol arg)	0.120 ± 0.008	0.129 ± 0.005
	Pentosidine (mmol/mol lys)	0.016 ± 0.003	0.020 ± 0.002

Data are mean ± SEM; at 20 weeks, *n* = 6 for both groups.

## Discussion

Given the potential ability of MG and MG‐derived AGEs to increase inflammation and reactive oxygen species and to modify intracellular and extracellular proteins, we had anticipated that the overexpression of GLO1 in endothelial cells, vascular smooth muscle cells, and macrophages would retard the development of atherosclerosis in diabetic *Apoe*^*−/−*^ mice in parallel with a reduction in kidney pathology. Likewise, we believed that knockdown of GLO1 would lead not only to kidney disease as we have previously shown (Giacco et al. 2014) but also increase atherosclerosis in nondiabetic *Apoe*^*−/−*^ mice. We observed, however, that elevated GLO1 activity in diabetic GLO1TG*Apoe*^*−/−*^ fails to impede diabetes‐induced accelerated aortic atherosclerosis in spite of reducing MG‐H1 immunoreactive proteins in aortic extracts. These same STZ‐treated GLO1TG*Apoe*^*−/−*^ mice are, nevertheless, protected against renal pathology and albuminuria. Furthermore, the reduced GLO1 activity in *Glo1*KD*Apoe*^*−/−*^ mice results in increased MG‐H1 immunoreactive proteins in the aorta but this does not exacerbate atherogenesis in nondiabetic mice at an age when *Glo1*KD mice manifest renal pathology and albuminuria similar to that seen in diabetic mice (Giacco et al. 2014). We have previously proposed that, in mice, MG levels determine the glycemic set‐point for the onset of diabetic nephropathy (Giacco et al. 2014) and this proposal is supported by the results of this study. In contrast to nephropathy, however, we now show that manipulation of GLO1 activity and as a consequence, dicarbonyl stress, does not alter the course of aortic atherogenesis in chow‐fed *Apoe*^*−/−*^ mice.

It is notable that while the GLO1 transgene reduced immunoreactive MG‐H1 in aortal extracts of STZ‐treated *Apoe*^*−/−*^ mice, it did not prevent increased dicarbonyl glycation (MG‐H1 at 20 weeks) of aortal extracellular matrix. The dichotomy between aortic immunoreactive MG‐H1 measured by immunoprecipitation/western blotting and collagen MG‐H1 levels measured by LC‐MS/MS may result from differences in the site of MG modification of the proteins and the origin of the MG. As collagen and other extracellular matrix proteins are likely poorly soluble in the extraction buffer used for analysis of immunoreactive MG‐H1, the MG‐H1‐modified proteins detected by this methodology may be primarily cellular proteins whose modification would be a function of intracellular MG levels and GLO1 activity. The MG‐H1 residues determined by LC‐MS/MS were selectively measured in the extracellular matrix. As turnover of extracellular matrix is relatively slow, its modification by MG may be largely an extracellular event. MG‐H1 residues have slow dynamic reversibility of approximately 12 days (Ahmed et al. 2002) and so decreased plasma MG is expected to decrease MG‐H1. The findings suggest that in the GLO1TG*Apoe*^*−/−*^ mice, decreased exposure of extracellular matrix to MG occurs only in the immediate locality of cells expressing the GLO1 transgene. MG has membrane permeability through passive diffusion of the unhydrated form (Rabbani and Thornalley 2012). Therefore, in the diabetic state, increased MG generated within cells likely produces increased release of MG from cells and contributes to AGE modification of extracellular proteins. In the transgenic model, it is expected that the maximum suppression of MG glycation is achieved for MG‐modified cellular proteins of cells where GLO1 activity is increased. Since MG that modifies the extracellular matrix likely originates from both cells that express the transgene and from those that do not, as well as from extracellular sources (Kalapos 2013), MG modification of extracellular proteins would be less susceptible to modulation of GLO1 activity than would MG modification of cellular proteins. The failure of the GLO1 transgene to moderate AGE modification of aortic extracellular matrix may contribute to its inability to impede atherogenesis.

The STZ‐induced increase in MG, CEL, and immunoreactive MG‐H1 in the kidney of *Apoe*^*−/−*^ mice was largely prevented by the human GLO1 transgene and this was associated with reduced mesangial sclerosis and albuminuria. Only a modest 36% increase of renal MG was found in STZ diabetic ApoE^−/−^mice with respect to nondiabetic controls. This may relate to regional changes in GLUT1 expression in the kidney in diabetes and related increased formation of MG by high cytoplasmic glucose concentration and increased anaerobic glycolysis. GLUT1 expression was decreased in the proximal tubular epithelium and increased in the cortical mesangial cells in experimental diabetes (Dominguez et al. 1994; D'Agord Schaan et al. 2001; Schaan et al. 2005). Increased kidney content of MG without concomitant increase in total protein MG‐H1 residues is suggestive of increased degradation of MG‐H1‐modified proteins to maintain proteome integrity. Without a related increase in expression this is likely to disturb the renal proteome. It is also notable that the increased MG‐H1 immunoreactivity in the STZ‐treated *Apoe*^*−/−*^ mice was largely confined to the glomeruli (Fig. 4G–J) as we had previously observed (Giacco et al. 2014). The increase in renal levels of 3‐NT, a marker of oxidative and nitrosative stress, following STZ adminstration that was seen in non‐TG*Apoe*^*−/−*^ mice was also moderated in the STZ‐treated GLO1TG*Apoe*^*−/−*^ mice. Levels of 3‐NT were not increased in the aortas of either STZ‐treated mice or in *Glo1*KD mice compared to their respective controls.

Our failure to observe an effect of increased GLO‐1 activity on atherogenesis could, in part, reflect the mouse model of diabetic atherosclerosis. As has been observed by others (Park et al. 1998; Forbes et al. 2004; Lassila et al. 2004), we found that induction of diabetes in *Apoe*^*−/−*^ mice with STZ resulted in an approximately two‐fold increase in apolipoprotein B‐associated plasma cholesterol. In human type 1 diabetic subjects, poor glycemic control is also associated with increased total and LDL cholesterol levels (The DCCT Research Group 1992; Petitti et al. 2007; Guy et al. 2009) although this is not as exaggerated as that which is seen in the diabetic *Apoe*^*−/−*^ mice. The hypercholesterolemia that occurs in the *Apoe*^*−/−*^ mice following STZ administration has been considered a shortcoming in this mouse model of human diabetic cardiovascular disease (Renard and Van Obberghen 2006; Hsueh et al. 2007; Kanter et al. 2007; Ramasamy and Goldberg 2010) and, in our case, may mask any macrovascular protective effects that are due to reducing dicarbonyl stress through overexpression of GLO1. *Apoe*^*−/−*^ mice also differ from humans in the distribution of cholesterol within the plasma lipoprotein subfractions. We have previously shown that an arginine residue near the amino terminus of apolipoprotein B is a preferred target of MG modification when LDL is exposed to concentrations of MG that are commonly observed in diabetic subjects. This apoB modification was shown to increase the affinity of LDL for proteoglycans and to favor LDL retention in the artery wall and thus potentially increase LDL atherogenicity (Rabbani et al. 2011). Our inability to demonstrate atheroprotection with GLO1 overexpression or increased atherosclerosis in *Glo1*KD mice may, in part, reflect that, in *Apoe*^*−/−*^ mice, cholesterol is primarily associated with remnant particles and not with LDL as is the case in humans.

Our finding that GLO1 overexpression can prevent diabetic albuminuria in STZ‐treated *Apoe*^*−/−*^ mice but not aortic atherosclerosis resembles, at least superficially, the situation in human type 1 diabetic patients where it has been shown that intensive glycemic control is very effective in reducing and in moderating the progression of diabetic nephropathy, retinopathy, and neuropathy (The Diabetes Control and Complications Trial Research Group 1993), whereas alleviation of cardiovascular disease through aggressive lowering of blood glucose levels requires longer follow‐up periods to become evident (The Diabetes Control and Complications Trial Research Group 1995; Nathan et al. 2005). This likely reflects the multifactorial etiology of atherosclerosis. While there is a general consensus that hyperglycemia has a direct, primary role in the development of diabetic microvascular disease, the importance of elevated glucose levels in the initiation and progression of diabetic atherosclerosis remains a subject of debate (Chait and Bornfeldt 2009; Orasanu and Plutzky 2009; Bornfeldt and Tabas 2011). Renard et al. (2006) showed that when low‐density lipoprotein receptor‐deficient (*Ldlr*^*−/−*^) mice were subjected to viral‐induced, autoimmune, pancreatic β‐cell destruction and maintained on a cholesterol‐free diet, they became hyperglycemic without significant changes in plasma lipid levels. The diabetic mice had more fatty‐streak lesions in the brachiocephalic artery with increased lesion‐associated AGE immunoreactivity compared to that in nondiabetic animals. Thus, in this model, hyperglycemia in the absence of changes in serum lipids was sufficient to elicit early atherosclerotic lesions. Renard et al. (2006) also showed that when the *Ldlr*^*−/−*^ mice were switched to cholesterol‐containing diets at the time of viral infection, the induction of diabetes resulted in higher serum cholesterol and triglyceride levels and, under these conditions, the altered serum lipids and not the hyperglycemia appeared to be primarily responsible for the increased atherosclerosis in the diabetic mice. This is likely also the case for the STZ‐treated *Apoe*^*−/−*^ mice in our study.

Agents that block formation of AGEs or break AGE cross‐links are being developed and tested for their abilities to avert complications in diabetic patients (Monnier 2003; Jandeleit‐Dahm and Cooper 2008). Forbes et al. (2004) showed no effect of aminoguanidine, an inhibitor of AGE formation and the thiazolium derivative, ALT‐711, a putative AGE crosslink breaker, on lesion size in the aortic arch of STZ‐treated *Apoe*^*−/−*^ mice maintained on a chow diet when analyzed 20 weeks after induction of diabetes, whereas in a separate study, both agents lessened renal pathology in diabetic *Apoe*^*−/−*^ mice (Lassila et al. 2004). Alt‐711 and aminoguanidine did, however, significantly reduce atherosclerosis in the thoracic aorta distal to the aortic arch and in the abdominal aorta (Forbes et al. 2004). The authors propose that, in these regions of the aorta, AGEs may make a major contribution to atherogenesis. In our study, we did not monitor atherosclerosis in the abdominal aorta, but we did quantify lesions by *en face* analysis in the descending thoracic aorta distal to the aortic arch and saw no difference in lesion area between STZ‐treated GLO1TG*Apoe*^*−/−*^ and STZ‐treated non‐TG mice. While both Alt‐711 and aminoguanidine lowered AGE immunoreactivity in tissues (Forbes et al. 2004), one cannot unequivocally attribute their protective effects to the reduction in AGEs as aminoguanidine is a known inhibitor of inducible nitric oxide synthase (Tilton et al. 2009) and ALT‐711 markedly attenuated the STZ‐induced increase in plasma cholesterol levels in the *Apoe*^*−/−*^ mice (Forbes et al. 2004). It is also possible that nonglyoxal/methylglyoxal‐derived AGEs such as N*ε*‐carboxymethyllysine (partly formed from glyoxal but mainly by the oxidative degradation of FL), whose formation is inhibited by AGE inhibitors (Forbes et al. 2004) but possibly not by GLO1 could participate in lesion development. More recently, ALT‐711 and another AGE inhibitor, pyridoxamine, were shown to retard the progression of established atherosclerosis in STZ‐treated *Apoe*^*−/−*^ mice, not only in the thoracic and abdominal aorta but also in the aortic arch (Watson et al. 2011).

In summary, by manipulating GLO1 activity in *Apoe*^*−/−*^ mice, we have demonstrated distinct differences in the diabetes‐induced metabolic changes that result in diabetic nephropathy and diabetic atherosclerosis, respectively. We confirm our previous findings on the essential role for increased MG levels in development of albuminuria and mesangial sclerosis in STZ‐treated mice, (Giacco et al. 2014) but now show that, in the same *Apoe*^*−/−*^ mice, increased GLO1 activity in endothelial cells, smooth muscle cells, and macrophages was insufficient to decrease MG glycation of aortal collagen and prevent the initiation and progression of atherosclerosis. Our findings also underline the decisive role for GLO1 activity in preventing hyperglycemia‐induced kidney damage. In the OVE26 mouse model of type 1 diabetes, it was shown that RAGE deficiency reduced glomerulosclerosis, improved renal function and this was accompanied by decreased MG and increased GLO1 expression (Reiniger et al. 2010). It was proposed that hyperglycemia provokes a positive feedback loop in which increased dicarbonyl stress induces RAGE expression which causes a downregulation of GLO1 expression resulting in impaired detoxification of dicarbonyls. This loop can presumably be broken either by disrupting RAGE signaling (Reiniger et al. 2010) or by increasing GLO1 activity (Giacco et al. 2014; and this study) with favorable effects on diabetic kidney disease. In this regard, it has also been reported that the angiotensin type 1 receptor blocker, candesartan, prevents angiotensin II‐induced downregulation of GLO1 and that this may contribute to its ability to protect against diabetic retinopathy (Miller et al. 2010). Thus, while dicarbonyl scavengers and AGE cross‐link breakers have long been considered as promising agents for prevention of diabetic complications, our results suggest that development of agents capable of increasing the activity of the glyoxalase system could be an attractive, alternative strategy to decrease diabetic kidney disease and, potentially, other microvascular complications. This might be achieved by exploiting the transcriptional regulation of GLO1 by Nrf2 (Xue et al. 2008; Rabbani and Thornalley 2012). Sulforaphane, an Nrf2 activator, has been shown to attenuate nephropathy in a mouse model of type 1 diabetes (Zheng et al. 2011). While GLO1 overexpression in vascular endothelial cells, smooth muscle cells, and macrophages failed to prevent accelerated atherogenesis in STZ‐treated *Apoe*^*−/−*^ mice, Nrf2 activators may prove to be more effective as the increase in GLO1 expression would be accompanied by a generalized increase in components of the antioxidant defense system.

### Limitations of the study

We have reported that the human GLO1 transgene under the control of the murine preproendothelin promoter is expressed in pure populations of both endothelial cells and smooth muscle cells isolated from aortas of GLO1TG mice (Vulesevic et al. 2014). It is possible, however, that in vivo, the preproendothelin promoter shows endothelial cell‐specific expression of transgenes as has been described previously (Harats et al. 1995; Ohashi et al. 1998; Bauer et al. 2005). Although no differences were detected between diabetic and nondiabetic mice in the level of GSH in heart tissue adjacent to the aortic sinus, we cannot exclude that GSH is, nevertheless limiting for GLO1 activity or that the oxidative stress within atherosclerotic lesions locally reduces GSH, dampening GLO1 activity and thus neutralizing the effects of the GLO1 transgene. Likewise, local oxidative stress could elicit a posttranslational modification of GLO1 resulting in its inactivation (Birkenmeier et al. 2010). While we did not see increased atherosclerosis in the *Glo1*KD*Apoe*^*−/−*^ mice which had similar serum lipid levels to wild‐type controls at 20 weeks of age, had we looked at older mice, it is possible that we would have seen that a more prolonged exposure to increased dicarbonyl stress in the aorta does result in more advanced atherosclerosis.

## Acknowledgments

We thank M. Hasu, L. Wilcox and C. Fournier for excellent technical assistance and C. Kennedy, (Kidney Research Institute, University of Ottawa) for the use of his metabolic cages.

## Conflict of Interest

No conflicts of interest, financial or otherwise, are declared by the author(s).
